# Quantifying the cumulative effect of low-penetrance genetic variants on breast cancer risk

**DOI:** 10.1002/mgg3.129

**Published:** 2015-01-14

**Authors:** Conor Smyth, Iva Špakulová, Owen Cotton-Barratt, Sajjad Rafiq, William Tapper, Rosanna Upstill-Goddard, John L Hopper, Enes Makalic, Daniel F Schmidt, Miroslav Kapuscinski, Jörg Fliege, Andrew Collins, Jacek Brodzki, Diana M Eccles, Ben D MacArthur

**Affiliations:** 1 Mathematical Sciences, University of SouthamptonSouthampton, SO17 1BJ, United Kingdom; 2 Cancer Sciences Academic Unit and University of Southampton Clinical Trials Unit, Faculty of Medicine, University of Southampton and University Hospital Southampton Foundation TrustTremona Road, Southampton, SO16 6YA, United Kingdom; 3 Human Genetics, Faculty of Medicine, University of SouthamptonTremona Road, Southampton, SO16 6YA, United Kingdom; 4 Centre for Molecular, Environmental, Genetic and Analytic Epidemiology, School of Population and Global Health, The University of MelbourneCarlton, Victoria, Australia; 5 Human Development and Health, Faculty of Medicine, University of SouthamptonTremona Road, Southampton, SO16 6YA, United Kingdom; 6 Institute for Life Sciences, University of SouthamptonSouthampton, SO17 1BJ, United Kingdom

**Keywords:** breast cancer, polygenic disorder, information theory

## Abstract

Many common diseases have a complex genetic basis in which large numbers of genetic variations combine with environmental factors to determine risk. However, quantifying such polygenic effects has been challenging. In order to address these difficulties we developed a global measure of the information content of an individual's genome relative to a reference population, which may be used to assess differences in global genome structure between cases and appropriate controls. Informally this measure, which we call relative genome information (RGI), quantifies the relative “disorder” of an individual's genome. In order to test its ability to predict disease risk we used RGI to compare single-nucleotide polymorphism genotypes from two independent samples of women with early-onset breast cancer with three independent sets of controls. We found that RGI was significantly elevated in both sets of breast cancer cases in comparison with all three sets of controls, with disease risk rising sharply with RGI. Furthermore, these differences are not due to associations with common variants at a small number of disease-associated loci, but rather are due to the combined associations of thousands of markers distributed throughout the genome. Our results indicate that the information content of an individual's genome may be used to measure the risk of a complex disease, and suggest that early-onset breast cancer has a strongly polygenic component.

## Introduction

Accumulating evidence suggests that many common diseases have a polygenic basis, in which large numbers of genetic variations combine with environmental and lifestyle factors to determine risk (Khoury et al. 2013). While genome-wide association studies (GWAS), and more recently exome and whole-genome sequencing projects, have found hundreds of genetic variants associated with disease, the ability to predict susceptibility from these associations is generally low because the contribution of individual variants to risk is often very modest. In the case of breast cancer, published GWAS have identified markers (single-nucleotide polymorphisms, or SNPs) in more than 70 independent regions (loci), the majority with odd ratios less than 1.1 (Bogdanova et al. 2013). Collectively these loci explain, in the statistical but not causative sense, approximately 15% of the familial relative risk which, when combined with the approximately 21% attributed to moderate- to high-penetrance variants (typically very rare mutations) in a dozen or so susceptibility genes, leaves almost two-thirds of the familial basis of the disease unaccounted for (Antoniou and Easton 2006; Bogdanova et al. 2013). It is likely that additional genes that explain a proportion of this missing heritability will be found using both whole-exome/genome and candidate gene sequencing of familial and young-onset cases, where the genetic component of risk is likely to be greatest (Hopper and Carlin 1992; Manolio et al. 2009; Park et al. 2012; Ruark et al. 2013; Akbari et al. 2014). Nevertheless, our current understanding of the genetic basis of breast cancer is still far from complete.

While most studies to date have focussed on individual genes or gene mutations and their contribution to disease, there has been limited effort to quantify the cumulative effect of variation across the whole genome on disease risk. This is partly due to the historical lack of sufficient data to appropriately quantify normal genomic variation within control populations, and the absence of the statistical techniques needed to analyze such large-scale variation. However, recent years have seen concerted effort to collect and collate the large numbers of genomes (for example the UK Department of Health's 100K initiative http://www.genomicsengland.co.uk) and there is now a need to develop the accompanying methodological tools to assess genomic variation (Yang et al. 2011; Zhou et al. 2013).

In order to begin to address this issue we describe here a measure of the extent to which a set of case genomes differ from a set of control genomes in their global structure. Our method uses ideas from information theory to provide a measure of the information content of an individual's genome with reference to a control population. The procedure first uses the reference population to estimate a probability measure on the space of all genomes, and then uses the estimated probability measure to assess how unusual an individual's genome is with respect to the reference population, as quantified by its self-information (also known in information theory as “surprisal”) (Cover and Thomas 1991). Formally, the resulting measure, which we refer to as the relative genome information (RGI), is the amount of information, measured in bits, required to specify the observed genome with respect to the unique encoding that minimizes the expected number of bits required to specify the genome of an individual drawn at random from the reference population. Informally, the RGI measures how unusual a genome is with respect to the reference population or, since we construct an information-theoretic measure closely related to the Shannon entropy, how “disordered” it is. Thus, someone with a higher RGI has a more unusual genome, either having less common alleles more often than expected, or having some particularly rare alleles. By contrast a lower RGI corresponds to having more common alleles more often, and therefore a less surprising genome.

We hypothesized that global measures of genome variation, such as RGI, might quantify the polygenic basis of complex diseases more completely than GWAS analyses that seek to find statistically significant associations of particular markers with disease. In order to test this hypothesis we compared the RGI of two independent samples of women with early-onset breast cancer genotyped for SNPs relative to three independent samples of unaffected controls.

## Methods

### Data sets and quality control

SNP genotypes obtained from blood samples from the following three independent studies were considered: (*i*) The Prospective study of Outcomes in Sporadic versus Hereditary breast cancer (POSH) cohort (Eccles et al. 2007). The POSH cohort consists of approximately 3000 women aged 40 years or younger at breast cancer diagnosis from which 574 cases were genotyped on the Illumina (San Diego, CA, USA) 660-Quad SNP array. Genotyping was conducted in two batches at the Mayo Clinic, Rochester, MN (274 samples) and the Genome Institute of Singapore, National University of Singapore (300 samples). A total of 536 samples that passed quality control filters were considered in this study (Rafiq et al. 2013). (*ii*) The Wellcome Trust Case Control Consortium (WTCCC, http://www.wtccc.org.uk/). The WTCCC consists of two independent sets of disease-free controls: 2699 individuals from the 1958 British Birth Cohort and 2501 individuals from the UK National Blood Service (NBS) Collection. Genotyping of both sets was conducted using the Illumina 1.2M chip. (*iii*) The Australian Breast Cancer Family Study (ABCFS) (McCredie et al. 1998; Dite et al. 2003). Cases were a subset of 204 of women aged 40 years or younger at breast cancer diagnosis from the ABCFS; controls were 287 unaffected women aged 40 years and older from the Australian Mammographic Density Twins and Sisters Study (Odefrey et al. 2010). Genotyping was conducted at the Australian Genome Research Facility using the Illumina 610-Quad SNP array. A summary of all data sets is given in Table1.

**Table 1:** Overview of case and control data sets

Data set	Size	Size after QC	Gender	Ethnicity	Genotyping platform
ABCFS cases	204	201	Female	Caucasian^1^	Illumina 610-Quad SNP array
POSH cases	574	536	Female	Caucasian^1^	Illumina 660-Quad SNP array
ABCFS control	287	280	Female	Caucasian^1^	Illumina 610-Quad SNP array
NBS control	2501	2501	Both	Caucasian	Illumina 1.2M chip
1958 control	2699	2699	Both	Caucasian	Illumina 1.2M chip

1 post-QC.

Only autosomes were considered and SNPs were excluded from each data set if they failed any of the following quality control filters: minor allele frequencies <1%; genotyping call rate <99%; significant deviation from Hardy–Weinberg equilibrium (*P* < 0.0001). All quality control filters were implemented using the software package PLINK (Purcell et al. 2007). In total, approximately 475,000 SNPs were genotyped in all five data sets. When comparing data sets and computing RGI only these shared SNPs were considered.

**Figure 1:**
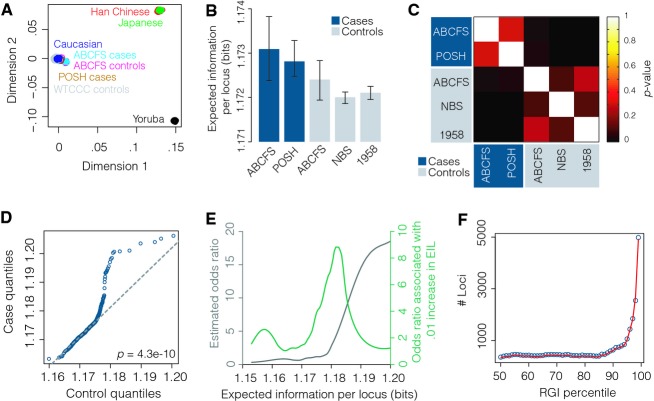
Breast cancer risk is associated with increased genome-wide disorder. (A) Multidimensional scaling plot of all samples and HapMap2 populations genotyped for ∼133,000 SNPs. (B) Expected information per locus (EIL) for each of the different data sets. Median ± 95% confidence intervals are shown. (C) Matrix of FDR adjusted *P*-values for comparisons of medians (two-sided Wilcoxon rank-sum test). (D) Q-Q plot of EIL in cases versus controls. *P*-value from a two-sample Kolmogorov–Smirnov test is shown. (E) Estimated odds ratio as a function of EIL. (F) Median number of loci required to account for the differences in EIL observed between cases and controls by percentile. 95% confidence intervals are within the markers, so are not shown.

Individuals with evidence of ethnic admixture were excluded by performing multi-dimensional scaling (MDS) analysis. Firstly, linkage disequilibrium (LD)-based pruning (*r*^2^ > 0.5) of genotypes was undertaken using PLINK to generate a reduced set of approximately independent SNPs. In total there were approximately 133,000 LD-pruned SNPs common to all samples. The HapMap data for the African, Asian, and Caucasian populations (Gibbs et al. 2003) were then used to provide reference population genotypes against which the genotype data of the cases and controls were compared (Fig.1A). We identified eight POSH and ten ABCFS samples that showed evidence of mixed ethnicity that did not cluster well with the HapMap Caucasian population reference sample, and these were excluded from further analysis. Since they only form a small subset of the total samples considered, the conclusions of our analysis do not differ without removal of these samples. However, we expect that, in general, significant ethnic variation within either the case or control populations would confound the results of our method.

### Quantifying relative genome information

Let *L* denote a set of locations in the genome (loci), and let Λ = {*A*, *C*, *G*, *T*} be the alphabet of possible alleles at each locus *l* ∈ *L*. Let Π_*l*_(*λ*, *μ*) denote the likelihood of finding the unordered allele pair (*λ*, *μ*) ∈ Λ × Λ at locus *l* ∈ *L* in the reference population and let Π be the product measure of Π_*l*_ over all *l* ∈ *L*. Thus, 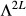 denotes the space of all possible genomes, and Π represents the probability measure on 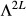. Now let *X *∈ Λ^2*L*^ be a genome with allele pair *X*_*t*_* *∈ Λ × Λ at locus *l* ∈ *L*. We define the relative local information (RLI) 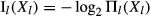 at each locus *l* ∈ *L* in the genome *X* and the RGI 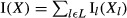 for each genome *X* of interest. For the purposes of comparison it is also convenient to normalize the RGI by *n*, the number of loci genotyped, to give the expected information per locus (EIL), 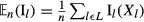. When comparing sequences of the same length the EIL and RGI are equivalent up to a normalizing factor. However, by normalizing by the number of loci sampled, the EIL allows comparison of relative information content of sequences of different lengths (for instance, comparison of relative information content of different chromosomes). The RLI is the natural information-theoretic measure of the “surprisal” of observing allele pair *X*_*l*_* *∈ Λ × Λ at locus *l* ∈ *L* given the probability measure Π_*l*_ (Cover and Thomas 1991). Similarly, the RGI is the natural information-theoretic measure of the “surprisal” of observing the genome *X*, given the probability measure Π.

In practice Π is not known a priori and must be estimated from an appropriate reference sample of similar ethnic background to that of the cases. Here, we estimated Π using the WTCCC 1958 birth cohort since it was the largest reference sample available. In all calculations, Π_*l*_ was estimated for each locus *l* ∈ *L* using all available genotypes in the reference population at that locus. Once Π had been estimated, the RGI was calculated for each genome in each of the remaining four (test) samples (POSH cases, ABCFS cases, ABCFS controls, NBS controls). The two additional independent sets of controls (ABCFS and NBS) were included in order to assess the robustness of the approximation of the background probability measure Π from the 1958 control cohort alone. For each of the four test samples, missing genotype data at each locus *l* ∈ *L* were assigned the expected value of Π_*l*_ (i.e., the Shannon entropy 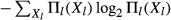 of Π_*l*_). This method of imputation minimizes the influence of missing data on the calculation of RGI. We also conducted all calculations using only those loci for which there were no missing readings in any of the data sets, and results obtained with and without imputation did not differ qualitatively. A brief worked example illustrating how Π was estimated, and the RLI and RGI were calculated, is given in the Data S1. Estimation of RGI for *N* case genomes takes *O*(*n*(*m* + *N*)) computational time, where *n* is the number of loci and *m* is the number of genomes in the control population, and can be conducted on a desktop PC for moderate sample sizes (thousands of samples and hundreds of thousands of genotyped loci).

### Statistical analysis

All analysis was conducted in *R* and Matlab (Natick, MA, USA) using custom written scripts. The association between EIL and disease odds was estimated using a logistic generalized additive model (Hastie et al. 2009). Tests for significant differences between groups were assessed using Wilcoxon rank-sum tests (two-sided tests were used when testing the null hypothesis of no difference in EIL between cases and controls against the alternative hypothesis that EIL differs in cases and controls; one-sided tests were used when testing the null hypothesis of no difference in EIL between cases and controls against the alternative hypothesis that EIL is raised in cases). All *P*-values were false-discovery rate (FDR) adjusted using the Benjamini and Hochberg (1995) procedure.

## Results

We did not observe any difference in EIL (RGI normalized by the number of loci genotyped, EIL) between the three different control sets (1958, NBS and ABCFS controls) indicating that the background measure Π was reliably estimated; similarly, no difference in EIL between the POSH and ABCFS cases was observed (Fig.1B and C). However, EIL was significantly higher in both the POSH and ABCFS cases than the three sets of reference controls (FDR adjusted *P* < 0.01, two-sided Wilcoxon rank-sum test) (Fig.1B and C). Since significant differences within case and control sets were not observed, we amalgamated samples to form one case set (consisting of the ABCFS and POSH cases) and one control set (consisting of the ABCFS, NBS and 1958 controls) for further analysis. Comparison of the distribution of RGI in amalgamated case set and amalgamated control set revealed significant differences in distribution structure (*P* = 4.3 × 10^−10^, two-sample Kolmogorov–Smirnov test) with the case distribution having a substantially heavier tail than the control distribution, indicating a greater proportion of samples with higher EIL (Fig.1D). To investigate further we conducted regression using a logistic generalized additive model (Hastie et al. 2009) in order to estimate the relationship between disease odds and EIL (Fig.1E). Consistent with the heavy-tailed nature of the case distribution we observed a strong positive association between odds ratio and EIL. In particular, the odds ratio increased sharply for EIL above 1.75, with the highest percentile EIL (above 1.183) having an odds ratio greater than 12 by comparison with the lower 99% (*P* < 1 × 10^−16^, Fisher's exact test). These results indicate that EIL is significantly elevated in breast cancer cases, with the highest percentiles EIL conferring a substantially increased risk.

In order to investigate the genetic basis for these observations we sought to assess whether the differences observed were associated with particular genomic loci or SNP annotations. We began by estimating the number of loci required to account for observed differences at each percentile using random resampling with replacement (1 × 10^4^ times) from the case genomes until the required difference was achieved. Differences in median EIL between cases and controls were found to be due to contributions from an estimated 327 distinct loci (median, 95% confidence intervals [306, 349]) (Fig.1F). The expected number of loci required to account for differences between cases and controls sharply increased with percentile, with differences in the 99th percentile (which conferred the greatest disease risk) requiring an estimated 4954 loci (median, 95% confidence intervals [4921, 5000]) (Fig.1F). These results indicate that observed differences in EIL are not due to high-penetrance variations at a small number of disease-associated loci, but rather are due to widespread variation at thousands of genomic loci.

**Figure 2:**
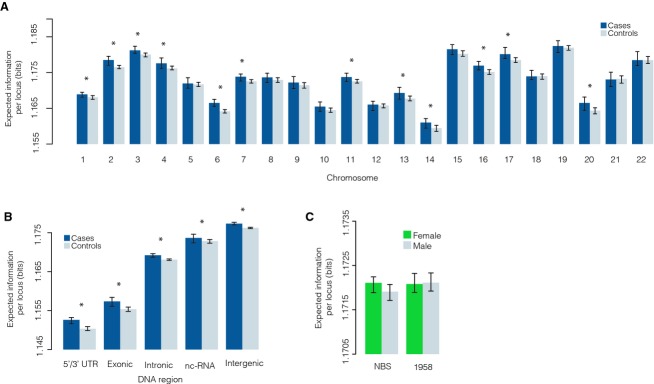
Disorder is not localized to specific regions of the genome. (A) Expected information per locus (EIL) by chromosome. (B) EIL by SNP annotation. (C) EIL in males and females in the controls. In all panels, median ± 95% confidence intervals are shown. Stars indicate significant changes at FDR adjusted *P* < 0.05 by one-sided Wilcoxon rank-sum test.

In order to investigate this further we assessed the EIL on individual chromosomes. We found that EIL was consistently elevated in the cases by comparison with the controls on 19 of 22 chromosomes (Fig.A), and significantly so on 12 of 22 chromosomes (FDR adjusted *P* < 0.05, one-sided Wilcoxon rank-sum test), indicating that differences in EIL are distributed throughout the genome. We also observed notable variations in EIL by SNP annotation, with the lowest EIL (and therefore the least variation within the samples) occurring in the 5′/3′ untranslated and exonic regions, and the highest EIL (and therefore the greatest variation within the samples) occurring in the intergenic regions (Fig.2B). This is consistent with previous assessment of relative mutation rates and suggests that 5′/3′ UTRs and exonic regions are subject to stronger negative selection than intergenic regions, in accordance with their phenotypic importance (Ward and Kellis 2012a,b; Khurana et al. 2013). In all annotation categories, we again observed a significant increase in EIL in the cases (FDR adjusted *P* < 0.05, one-sided Wilcoxon rank-sum test) (Fig.2B). These results indicate observed differences in EIL are not localized to distinct regions of the genome (either chromosomes or SNP annotations) but rather are due to widespread variation distributed throughout the genome.

## Discussion

Genetic factors that contribute to breast cancer risk range from rare highly penetrant functionally deleterious mutations in genes like BRCA1 and BRCA2 to genetic variants that are relatively frequently observed and are associated with small increases in risk (Mavaddat et al. 2010). However, we do not yet have a complete understanding of the genetic basis of breast cancer. Much of the missing heritability may be either very rare highly penetrant genes not currently known or, more likely, hundreds to thousands of rare genetic variants with small effect sizes. Current approaches to discovering low-penetrance genetic susceptibility alleles using GWAS rely on risk alleles being relatively common in the population. Even with case–control studies involving hundreds of thousands of individuals, identifying all the genes responsible for susceptibility is likely to prove difficult if important effects relate to the accumulation of rare low-penetrance alleles. By comparing individual genetic sequences with that expected from a control population our approach assesses the cumulative effect of low-penetrance alleles on disease risk. Our results suggest that such cumulative effects are a significant component of the missing heritability in breast cancer. Prior to analysis all genotyping data were subjected to stringent quality assurance and we observed no association between sex, sequencing platform, time/place of sequencing and EIL, indicating that poor data quality or variation in genotype due to ethnicity or sex are unlikely to explain our results (Figs.1B, C, and 2C). Rather, changes in EIL appear to quantify statistically significant differences in allele frequencies between breast cancer cases and controls.

Taken together our analysis indicates that early-onset breast cancer has a strongly polygenic component, involving variation at thousands of markers distributed throughout the genome. Thus, along with assessment of known risk-associated variants, the information content of an individual's genome is likely to be a useful predictor of breast cancer susceptibility. Further analysis of the relationship between global genome structure and disease risk may reveal a similarly polygenic basis for a variety of other complex diseases.

## Conflict of Interest

None declared.
