# Supplementary material for: Quantifying the cumulative effect of low-penetrance genetic variants on breast cancer risk
Source: Mol Genet Genomic Med. 2015 Jan 14;3(3):182–8. doi: 10.1002/mgg3.129 (PMC4444159; doi:10.1002/mgg3.129)
Supplement: Supplementary file 1 — Data S1. Supplementary Materials. [file mgg30003-0182-sd1.docx]

**Supplementary Materials**

## A worked example

Consider sampling 5 individuals from an unknown background population. In this illustrative example there are 2 letters in the alphabet $\Lambda=\left\{ A,B \right\}$ and at a particular locus the following alleles are observed:

|  | Individual 1 | Individual 2 | Individual 3 | Individual 4 | Individual 5 |
| --- | --- | --- | --- | --- | --- |
| Locus 1 | AA | AA | AA | BB | BA |

To estimate the true probability of finding each allele pair in the background population we count the frequencies of the alleles A and B to obtain:

$$p(A)=\frac{7}{10}, p(B)=\frac{3}{10}.$$

We may then estimate the Hardy-Weinberg proportions:

$$\Pi_{1}\left( AA \right)\approx p\left( A \right)\times p(A)=\left( \frac{7}{10} \right)^{2},$$

$$\Pi_{1}(BB)\approx p(B)\times p(B)=\left( \frac{3}{10} \right)^{2},$$

$$\Pi_{1}\left( AB \right)=\Pi_{1}(BA)\approx2\times p(A)\times p(B)=2\times\left( \frac{7}{10} \right)\times\left( \frac{3}{10} \right).$$

The RLI for each pair is found by taking the negative logarithm of these probabilities:

$$-\log_{2}(\Pi_{1}(AA))=1.0291,$$

$$-\log_{2}(\Pi_{1}(BB))=3.4739,$$

$$-\log_{2}(\Pi_{1}(AB))=1.2515,$$

where logarithms are taken to base 2 so that information is measured in bits. Since the pair BB is rare in the population it imparts more information whenever it is observed; by contrast since the pair AA is common, it imparts less information whenever it is observed.

Suppose now that a second locus is also sequenced and the following alleles are observed:

|  | Individual 1 | Individual 2 | Individual 3 | Individual 4 | Individual 5 |
| --- | --- | --- | --- | --- | --- |
| Locus 2 | AA | BA | BA | BB | AA |

A similar calculation yields the RLI of the second locus and the RGI for each individual (here, the ‘genome’ has just 2 loci):

|  | Individual 1 | Individual 2 | Individual 3 | Individual 4 | Individual 5 |
| --- | --- | --- | --- | --- | --- |
| RLI 1 | 1.0291 | 1.0291 | 1.0291 | 3.4739 | 1.2515 |
| RLI 2 | 1.4739 | 1.0589 | 1.0589 | 2.6439 | 1.4739 |
| RGI | 2.5030 | 2.0880 | 2.0880 | 6.1178 | 2.7254 |

In this case, individual 4 has the least common alleles at both loci, and correspondingly has the largest RGI. Individuals 2 and 3 have the most common alleles are therefore the lowest RGI. In this calculation the five individuals form the control population from which the probability measure $\Pi$ on $\Lambda^{2L}$ is approximated. Once this measure has been approximated, it may be used to assess the RGI of unseen case genomes. For example, a case individual with the genome [AA, BB] has RGI = 1.0291 + 2.6439 = 3.673.
